# First statement on preparation for the COVID-19 pandemic in large German Speaking University-based radiation oncology departments

**DOI:** 10.1186/s13014-020-01527-1

**Published:** 2020-04-07

**Authors:** Stephanie E. Combs, Claus Belka, Maximilian Niyazi, Stefanie Corradini, Steffi Pigorsch, Jan Wilkens, Anca L. Grosu, Matthias Guckenberger, Ute Ganswindt, Denise Bernhardt

**Affiliations:** 1 Department of Radiation Oncology, Technical University of Munich (TUM), Klinikum rechts der Isar, Ismaninger Straße 22, D-81675 München, Munich, Germany; 2 Department of Radiation Sciences (DRS), Institute of Radiation Medicine (IRM), Ingolstädter Landstraße 1, Neuherberg, Germany; 3 Deutsches Konsortium für Translationale Krebsforschung (DKTK), Partner Site Munich, Munich, Germany; 4 Department of Radiation Oncology, University Hospital, LMU, Munich, Germany; 5 Department of Radiation Oncology, Medical Center, University of Freiburg, Freiburg im Breisgau, Germany; 6 Deutsches Konsortium für Translationale Krebsforschung (DKTK), Partner Site Freiburg, Freiburg im Breisgau, Germany; 7 Department of Radiation Oncology, University Hospital Zurich, University of Zurich, Zurich, Switzerland; 8 Department of Radiation Oncology, Medical University of Innsbruck, Innsbruck, Austria

**Keywords:** COVID-19, Fractionation, Tandem-teams, Pandemic, Radiotherapy

## Abstract

The COVID-19 pandemic is challenging modern radiation oncology. At University Hospitals, we have a mandate to offer high-end treatments to all cancer patients. However, in times of crisis we must learn to prioritize resources, especially personnel. Compromising oncological outcome will blur all statistics, therefore all measures must be taken with great caution. Communication with our neighboring countries, within societies and between departments can help meet the challenge. Here, we report on our learning system and preparation measures to effectively tackle the COVID-19 challenge in University-Based Radiation Oncology Departments.

## Introduction

There is a nationwide concern about the coronavirus disease 2019 (COVID-19) and immediate medical emergency caused by the infection with severe acute respiratory syndrome coronavirus 2 (SARS-CoV-2); currently, this virus is the largest global public health threat leading to a major challenge of medical systems in all countries. The family of *Coronaviridae* consists of a group of large, single, plus-stranded RNA-viruses which have been isolated from several species; common symptoms in humans include common cold and diarrheal illness [1]. Almost 17 years ago, in China, a new coronavirus termed Severe Acute Respiratory Syndrome Coronavirus (SARS-CoV) caused the SARS outbreak and, within weeks, spread to more than a dozen countries on several continents including Asia, Europe, North and South America. The crisis affected over 8000 humans and lead to around 800 deaths [2].

As early as 2015, an international research group from USA, Switzerland, and China reported on the pathogenic potential of SARS-like Coronaviruses (CoVs) which were at that time circulating in Chinese horseshoe bat populations [3]; the group extensively described that these viruses can replicate effectively in primary human airway cells, and demonstrated the potential to affect the mouse lung and lead to considerable pathogenesis. Alarmingly, their experiments showed that available SARS-based therapeutics, including immunotherapeutics, vaccines, antibodies and other prophylactic modalities failed to neutralize and protect from CoVs. While elucidating the pathogenic potential, the authors warned of cross-species transmission.

Only 5 years later, this virus has become a lethal treat for humans; in December of 2019, Wuhan was the starting point of a worldwide pandemic, which supposedly originated from Wuhan’s wild animal market [4–6]. At the end of January 2020, the first case of a positively tested patient was reported in Germany in Munich, Bavaria, and according to the current status as of March 22, 2020, 18,610 cases were reported in Germany, including 55 deaths (https://www.rki.de/DE/Content/InfAZ/N/Neuartiges_Coronavirus/Fallzahlen.html).

Radiation Oncology (RO) is a key discipline in oncology and currently more than 50–60% of all cancer patients are treated with radiotherapy at some point of their disease [7]. Most curative treatment regimens include radiotherapy, many sequentially with surgery, chemotherapy, or as concomitant treatments. Albeit the fear of COVID-19 is currently crossfading all other illnesses, it is the mission of radiation oncologists and other cancer care physicians to courageously take sides for our cancer patients.

We are now faced with increasing hygiene measures, more complicated and difficult treatment procedures aggravated by facial masks and personal protection equipment [8]. The number of employee illnesses is increasing and jeopardizing our efforts to secure continuous patient treatments. While most university-based radiation oncology departments are equipped with high-end radiotherapy devices which offer innovative radiation treatments on a high and evidence-based level, human resources are the most precious asset in the COVID-19 challenge.

Over the last years, independently of any pandemic scenario, there has been a wave of arguments that foster hypofractionated treatments in radiation oncology. Most societies actively supporting those concepts are clearly driven by a force of limited treatment capacity paired with a constricted medical system; this forefront is most actively driven by physicians in the United Kingdom (UK). Others have followed, and in indications such as breast cancer [9] and, now following, prostate cancer, concepts of hypofractionation are spreading [10]. While certain low-risk indications might not be undertreated, there is a quiet word of caution for high-risk, fast growing tumors and other factors or tumor biology and normal tissue, that most likely will lead to undertreatment with hypofractionated regimens [11]. In metastatic disease, the argument for hypofractionation is clearly driven by the reduced survival times of palliative patients; however, lessons learned from the treatment of vertebral metastases have nicely shown that short hypofractionated treatments such as 5 × 4 Gy or 1 × 8 Gy for vertebral metastases may help for short term pain reduction, but longer-term local control is significantly lower than with 10–13 × 3 Gy, and even higher with 40Gy in 2 Gy fractions [12–18]. Estimating life expectancy is always controversial, even among advanced health care providers and with the help of high-end diagnostic, molecular markers and prognostic scores, [19–25].

Now, in our world of high-end radiation oncology service, we are facing a new challenge: keeping our radiation oncology service up and running during the COVID-19 challenge. In spite of specialized and enforced hygiene measures, the crisis has triggered every unit to activate and improve emergency scenarios. Considering potential limitations of personnel capacity, all emergency measures will include, at a certain point, hypofractionated and very pragmatic fractionation schedules. These should ideally be evidence based. However, all measures must be associated with the appropriate word of caution not to compromise oncological outcome if not ultimately necessary due to collapsing resources.

Preparing our radiation oncology departments should therefore follow a series of regulations and measures, to ensure high-end oncological treatment as long as possible.

Overall, ***personell, patient and device hygiene*** are the most important measure on wards, radiotherapy units, chemotherapy treatment rooms, administration areas, offices as well as all other public areas. Adequate disinfectants must be provided, at the entrance to the hospital to the ward, to the radiotherapy unit etc., and patients as well as staff must be educated on the effective use of all disinfectants used. For superficial disinfectants, it is important to use quickly active solutions so the radiotherapy scheduling does not come to a standstill. One critical issue in this special pandemic situation is the availability of protective clothing. As long as sufficient numbers of protective gear are available, surgical masks should be worn according to the indications continuously updated by the World Health Organization (WHO) [26], the Robert Koch Institute (RKI) [27], as well as the respective hospital standard operating procedures (SOP). Staff and patients must be advised that masking protects both sides. The lesson of not protecting the medical staff from the very beginning can be drawn from the dramatic Italian experience. The medical staff has become not only a casualty itself, but also a potential source of infection that is transmitted to every patient they meet and treat [28, 29]. In cases of SARS-CoV-2 positivity, a more advanced personal protection equipment is needed and includes: disposable overalls (tunics and/or trousers), disposable gowns and eye protection should be used. FFP2 masks and overshoes are not recommended in all institutions and countries. Visits of relatives and accompanying people should be stopped as early as possible to departments dealing with cancer patients [30].

- If the resources allow it, cancer patients, especially those undergoing chemotherapy or those with immunosuppression should wear adequate protective masks. A recent analysis from Wuhan, published in Lancet Oncology revealed that patients with cancer might have a higher risk of COVID-19 than individuals without cancer. Additionally, the study showed that patients with cancer had poorer outcomes and more rapid deterioration from COVID-19 [31, 32]. The authors even concluded, that chemotherapy should be postponed if possible.
- In hospitals where there is no central triage unit, it is recommended to carry out a triage at the entrance of the radiotherapy facilities for the verification of symptomatic patients or the evaluation of contacts to SARS-CoV-2 positive patients in everyone accessing the radiotherapy areas. Standardized questionnaires and measuring the body temperature are advisable. Furthermore, outpatients may be called by telephone 1 day in advance to their appointments to screen for symptoms and ask for contacts to SARS-CoV-2 positive patients/recent stays in so called “risk areas”.
- Firstly, the ***management team*** of the Department of Radiation Oncology must be clearly identified and the executive power of each member of the management team must be clarified and communicated to the entire team in this special situation. Secondly, the management team must be divided into a ***tandem operation team (ideally 50% on site, 50% off site)*** to allow for backup solutions. Through this measure it can be secured that the executive force of the Department is not compromised throughout the crisis. The management team should communicate via teleconferencing systems on a regular basis to communicate all details and exchange any changes of situation and not meet in person.
- The staff of the Department of Radiation Oncology outside of areas ultimately relevant for the retention of clinical department operations should be limited. The offices, research areas, wards as well as other clinical areas of the department should be restricted to people working **in system-critical areas**. The personnel may include staff for the basic operations of the Department, including, in particular, indispensable employees in administration and patient care. For research areas, specific institutional guidelines can be released. In some centers, critical persons include personnel involved in animal husbandry and animal research facilities, as well as the supervisors of scientific long-term experiments and technical infrastructure. In other scenarios, research animal facilities are generally closed and all staff is moved towards maintaining clinical infrastructure and operations, as needed. The management team should strictly supervise and coordinate these measures and enable home-office possibilities.
- For all ***system-relevant areas, a tandem-staffing*** must be planned at the earliest possible timepoint. Tandems comprise two different persons who work as back up for each other. They are not supposed to be in physical contact, they are not allowed to enter the same room at the same time. Importantly, communication between tandems has to be ensured at all time by digital means to maintain tandem members at the same level of information. These tandem teams should be built especially for doctors, with special focus on the group of radiation protection physicians to comply with regulatory responsibilities at any timepoint of the crisis. Also, technicians, medical physics experts (MPEs), radiation safety officers as well as secretaries and nurses should be places in tandem teams who stand in continuous mutual consultation. These tandem groups regularly switch on- and off-site assignments every fortnight to overcome the 14-day half-life of the SARS-CoV-2. Theses tandem groups depend on the size and resources of the department, however, even subgroup tandem-staffing could be a compromise solution.
- Some institutions recommend that all other staff who is **not working in system-critical areas** should work in home office, if possible. Special assignments for the off-site work will be distributed by the management team, the group leaders or other supervisors. All off-site workers must be available to supervisors as well as colleagues via landline, mobile phone or e-mail. However, if this is not possible and on-site working is performed, social distancing and special hygiene requirements are essential. Digital communication should be evaluated were possible.
- In order to meet the special challenges of these days, management and the works council of some institutions have reached an agreement on the subject of “***confidential working hours***”, which makes the current situation easier for both employers and employees. This means, the employer is not actively checking working hours, and at some institutions electronic time recording is de-activated since many individuals will be working off-site or in home office situations.
- All planned leave days of personnel essential for clinical operations should be cancelled during the team of crisis. This ensures that essential workforce is present and can be recruited to the department as necessary. Another option is to remain on stand-by, for immediate deployment when other personnel is absent. In all other groups of the workforce not essential for clinical operations staff may be asked to take all vacation days possible during this time.
- Conferences and interdisciplinary tumor boards should be switched to ***digital solutions*** like email and video conferences. If this is not possible, only relevant staff with executive functions or experts should participate to minimize group size and exposure in order to prevent viral spread between staff and different departments.
- Interdisciplinary case discussions (tumorboards) are a standard in oncology. In these special times, these discussions should not be compromised. Rather, these conferences should be strengthened and intensified to find the optimal solution for each patient together with the neighboring disciplines, such as hematology/oncology and surgery.
- All ***follow-ups should be critically evaluated and postponed*** if not regarded essential. Outpatient clinics are advised to use online, digital or medical counselling by phone. However, it is important to secure appropriate ***identification and treatment of critical cases***. For all outpatient clinics as well as necessary follow-ups, entry point screening is necessary; patients should fill out a hygiene risk questionnaire asking for possible symptoms and potential residency or travel history to risk areas.
- In order to minimize the risk for both the patients and health care staffing by the repetitive risk of exposure, the indications for radiotherapy must be strictly defined. Table 1 gives an evidence-based overview of possible hypofractionated regimens or observational strategies for a variety of entities which can be considered during this crisis. The potential benefits and risks of altered fractionations should be carefully discussed with the patient. Procrastinating certain pathologies by evaluating the risk/benefit ratio in each individual case is advisable. Moreover, benign diseases should not be treated at all. **Importantly, this table does not represent standard-of-care regimens and the fractionations should not be used routinely outside of special crisis situations.**
- In times were surgical and anesthesiological capacity might become even more rare, **the equieffectivity of radiotherapy and radiochemotherapy regimens as non-invasive treatments should be discussed**. For selected indications, literature is summarized in Table 2, 3 and 4.
- Prepare staff and patients how to best mitigate the impact of a 2- to 3-week interruption in treatment. From the aftermaths from hurricane Maria in Puerto Rico there are rough ASTRO guidelines based on the limited evidence available [73–76].
- If possible members of the staff at high risk for severe COVID-19 courses of disease should be identified and, if possible from an organizational point of view, allotted to off-site assignments to allow for optimal social distancing and staff protection. This may include older employees, those with comorbidities or other factors.

**Table 1:** Evidence-based recommendations for fractionated-adapted pandemic radiation oncology

Site	Criteria	Concept	Evidence/ Guideline
**Glioblastoma**	*KPS 100–80; age > 65/60 years*	2.67Gy/ 40.05 Gy + TMZ (MGMT methylated)	Perry et al., 2017 [33]
	*KPS < 60*	5.0 Gy / 25.0 Gy, no TMZ	Roa et al. JCO 2005 [34]
	*KPS < 50; age > 70 years*	TMZ mono (MGMT methylated) or BSC	Malmström et al. 2012 Lancet Oncology [35]
	*All age groups, good performance status*	Tumor treating field, especially if TMZ is postponed due to pandemic risk for severe pneumonia	Stupp et al. JAMA 2015 [36]
**Brain metastases**	*1–10 BM; good performance status*	Stereotactic radiosurgery 1 × 18 Gy, or 1 × 20 Gy	Kocher et al. JCO 2011 [37] Yamamoto et al. Lancet Oncology 2017 [38]
	*Postoperative*	SRS of resection cavity e.g. 7 × 5 Gy or single fraction	Brown et al. Lancet Oncology 2017 [39] Mahajan et al. 2017 [40] Sahgal et al., 2017 [41]
	*Driver mutations*	ALK: Targeted therapy first	various
	*Life expectancy > 3 months*	5 × 4 Gy Whole Brain Radiotherapy (WBRT)	Borgelt et al. RED Journal 1981 [42]
	*Poor performance status*	Evaluate BSC with critical view of steroids	Mulvenna et al. Lancet 2016 [43] NCCN Guidelines
**Meningeoma**	*WHO°1*	Watchful waiting or 5 × 5 Gy	NCCN Guidelines Alfredo et al. 2019 [44]
	*WHO°2*	Watchful waiting after complete resection	NCCN Guidelines EANO Goldbunner et al. Lancet Oncology 2016 [45] RTOG 0539 Rogers et al. J Neurosurg 2018 [46]
**Breast**	*DCIS*	Omission of RT in low risk DCIS or Active surveillance + endocrine therapy or 15 × 2.67/ 40.05 Gy	Nilsson et al. Radiother Oncol 2015 [47]
	*Invasive Breast cancer*	Omission of RT in low risk carcinomas or 15 × 2.67Gy/ 40.05Gy or 5 × 5.2 /26Gy	Haviland et al. Lancet Onc. 2013 [48] FAST Forward Trial [49, 50]
	*Including lymphatic drainage*	15 × 2.67Gy/ 40.05Gy	Haviland et al. Lancet Onc. 2013 [48]
	*Postmastectomy*	Hypofractionation if no implant, 15 × 2.67Gy/40.05Gy or 15 × 2.9/43.5 Gy	Wang et al. 2019 [51]
	*Partial breast*	ASTRO PBI criteria 38.5 Gy/ 10 fx BID 30 Gy/ 5 fx daily 28.5 Gy/ 5 fx once weekly 26Gy / 5 fx daily 20 Gy/ 1 fx IORT	Correa et al., 2017 [52] Livi et al. Eur J of Cancer 2015 [53] Brunt et al. FAST Forward Trial 2016 [49, 50] Vaidya et al. Lancet 2014 [54] Veronesi et al. Lancet Onc 2014 [55]
**Lung**	*NSCLC Stage I*	SBRT e.g. 3 × 15 Gy, 8 × 7.5 Gy, 1x34Gy [56]	Guckenberger et al. J Thoracic Onc 2013 [57]
	*NSCLC stage III*	24 × 2.75 Gy	DOI: 10.1016/j.ejca.2006.09.005
	*SCLC limited disease*	15 × 2.67Gy / 40.05 Gy	Sculier et al. Annals onc 2008 [58, 59]
**Prostate**	*Low risk*	Postpone Therapy perhaps with ADT, active surveillance or hormonotal deprivation	NCCN Guidelines
	*Active Surveillance*		Hamdy et al. & Donovan et al. NEJM 2016 ProtecT [60]
	*Watchful waiting*	Life expectancy < 10 years, T1–4, GS ≤7	NCCN Guidelines
	*Intermediate Risk or high Risk*	neoadjuvant ADT 2–3 months	DART01/05 GICOR Zapatero et al. Lancet Oncol 2015 [61] EORTC 22991 Bolla et al. JCO 2016 [62]
		20x 3Gy / 60Gy	CHHIP Dearnaley et al.,2016 and 2017 [63, 64]
		age < 75 years: 42.7 Gy/ 7 Fx every other day	HYPO-RT-PC, Widmark et al., [65]
	*Adjuvant/ Salvage Situation*	Watchful waiting or ADT	NCCN Guidelines
		52.5 Gy / 20 fx	Chin et al. RED Journal 2020
	*Lymphatic drainage RT*	Evaluate critically, only if visible nodal disease	GETUG-01-Trial Pommier et al., J Clin Oncol 2007 and IJROBP 2016 Supiot et al. 2013 [66–68]
**Palliative setting**	*Bone metastases*	8 or 10 Gy/ 1 fx 20 Gy/ 5 fx 21 Gy/ 3 fx	Chow et al. JCO 2007 [69]
	*Head & neck*	*QUADshot:* 3.5 Gy BID × 2 days, repeated Q4 weeks interval × 2 times	Spanos et al. Int J Radiat Oncol Biol Phys. 1989 [70]
	*Bleeding*	8 Gy / 1 fx	Sapienza et al. Clinical and Translational Radiation Oncology 2019 [71]
	*Oligometastatic*	SBRT, e.g. 1–5 fractions	Otake et al. Cancers 2019 [72]

*KPS* Karnofsky performance status, *TMZ* temozolomide, *BSC* best supportive care, *BM* brain metastases, *ADT* androgen deprivation therapy

**Table 2:** Esophageal Cancer. Neoadjuvant Therapy plus surgery vs. surgery versus definitivie Radiochemotherapy

Kranzfelder et al. Br J Surg 2011	Metaanlysis Nine RCTs involving neoadjuvant CRT versus surgery, eight involving neoadjuvant chemotherapy versus surgery, and three involving neoadjuvant treatment followed by surgery or surgery alone versus dCRT	Neoad. RChT: Sign. OS-Benefit (HR 0,81) Neoadj. ChT: No OS-Benefit (HR 0,93, *p* = 0,36) No OS difference after dCRT demonstrated a significant survival benefit, but treatment-related mortality rates were lower (HR 7·60, *P* = 0·007) than with neoadjuvant treatment followed by surgery or surgery alone.
Stahl et al. JCO 2005	Phase III-Study (1994–2002, 189 Pat.) CRT: 40 Gy + Cisplatin/Etoposid vs. def. RCHT	adding surgery to chemoradiotherapy improves local tumor control but does not increase survival of patients with locally advanced esophageal SCC. Tumor response to induction chemotherapy identifies a favorable prognostic group within these high-risk patients, regardless of the treatment group.
FFCD 9102 Bedenne et al. JCO 2007	Phase III-Study (1993–2000, 259 Pat.) T3 N0–1, 89% SCC Neoadj. CRT: Split course 30 Gy in 3 Gy or 45 Gy in 1,8 Gy + 2x Cisplatin/5-FU Random.: OP vs. def. CRT	two-year survival rate was 34% in arm A versus 40% in arm B (hazard ratio for arm B v arm A = 0.90; adjusted *P* = .44). Median survival time was 17.7 months in arm A compared with 19.3 months in arm B Author conclusion: there is no benefit for the addition of surgery after chemoradiation compared with the continuation of additional chemoradiation

**Table 3:** Radiotherapy/Radiochemotherapy for Rectal Cancer

Maas, JCO, 2011	Patients with a cCR after CRT were prospectively selected for the wait-and-see policy with magnetic resonance imaging (MRI) and endoscopy plus biopsies prospective cohort study 21 patients	control group: FU 35 Mo, 2-J-DFS 93%, 2-J-OS 91% control group consisted of 20 patients with a pCR after surgery who had a mean follow-up of 35 ± 23 months. For these patients with a pCR, cumulative probabilities of 2-year disease-free survival and overall survival were 93 and 91%, respectively.
Habr-Gama, IJROBP 2014	183 Pat., cT2–4 or N+, CRT (50–54 Gy + 5-FU), Response after 8 weeks, patients with cCR were enrolled in a strict follow-up program with no immediate surgery (Watch and Wait).	Local recurrence may develop in 31% of patients with initial cCR when early regrowths (≤ 12 months) and late recurrences are grouped together. More than half of these recurrences develop within 12 months of follow-up. Salvage therapy is possible in ≥90% of recurrences, leading to 94% local disease control, with 78% organ preservation.
OnCoRe Renehan, Lancet Oncol 2016	129 Pat., RChT, if cCR no surgery	38% 3 J LR, 88% Salvage-OP, better colostomy-free survival (74% vs 47%) in R T group A substantial proportion of patients with rectal cancer managed by watch and wait avoided major surgery and averted permanent colostomy without loss of oncological safety at 3 years.
Appelt et al. Lancet Oncol 2015	prospective cohort study (2009–2013, 51 Pat.), Follow up 29 months CRT with 50 Gy incl. SIB 60 Gy + HDR-Brachy 1 × 5 Gy + Tegafur-Uracil 300 mg/m2	40 Pat. With cCR (78%) Local recurrence in the observation group at 1 year was 15·5% (95% CI 3·3–26·3). The most common acute grade 3 adverse event during treatment was diarrhoea, which affected four (8%) of 51 patients. Sphincter function in the observation group was excellent, with 18 (72%) of 25 patients at 1 year High-dose chemoradiotherapy and watchful waiting might be a safe alternative to abdominoperineal resection for patients with distal rectal cancer.
Garcia-Aguilar et al. Lancet Oncol 2015 ACOSOG Z6041 29	Phase II-study (77 Pat.), FU 52 Mo. cT2 N0 < 4 cm (EUS oder MRT) neoadj. CRT 50–54 Gy + Oxaliplatin/Capecitabine after 4–8 weeks local excision for patients with stage T2N0 rectal cancer.	3-year DFS 88% 49% ypT0/is, 14% ypT1, 31% ypT2, 4% ypT3

**Table 4:** Stereotactic Body Radiotherapy (SBRT) vs. Surgery for lung cancer

ROSEL/ STARS Chang (Lancet Oncol 2015)	58 Pat. 2 rand., prosp. Studies T1–2 N0, < 4 cm 54 Gy in 3 Fx (peripher) o. 50 Gy in 4 Fx (zentral) vs. surgery	SBRT vs. surgery 3y-OS: 95 vs. 79% (s) LC: 94% vs. 100% (n.s.) >°III Tox: 10 vs 44%
Zheng et al. IJROBP 2014	Metanalyse, 40 studies of SBRT and 23 Studies with surgery St. I NSCLC Median Age 74 J. vs. 66 J.	5-year OS 40% vs. 66% (lobectomie) vs. 71% (sublobectomie)
Stokes et al. JCO 01/2018 30 & 90 day mortality	National Database, 76,623 patients OP (78% lobectomy, 20% sublobar resection, 2% pneumonectomy) vs. 8216 patients SBRT Propensity score matching	surgical mortality rates were significantly higher with increased extent of resection and age at 30 days, 2.41% vs 0.79% (s), 90 days, 4.23% vs 2.82% (s) with matched pairs

As long as possible, the provision of high-end oncological care should be maintained. The despair of several Italian and Spanish Radiation Oncology Departments in the emerging COVID-19 crisis argued for triage and extensive application of hypofractionated and ultimately ultra-hypofractionated radiation regimens [8, 77]. Although the overall pandemic situation might force individuals for this strategy, it must be always kept in mind that oncological care should not be compromised if not ultimately needed, especially in the curative setting. Additionally, all statistics of survival will be massively blurred if broad radiation oncology service is restricted and COVID-19 fear and pandemic crisis force us to use minimalistic radiation efforts.

While most recommendations from other radiation oncology societies are in line with the above-mentioned measures during the COVID-19 outbreak, the treatment of COVID-19 positive patients remains the greatest challenge.

- A continuous triage evaluation is needed to detect an early onset of typical symptoms of COVID-19 (fever, cough, sore throat, shortness of breath, fatigue) in patients already receiving treatments at the Radiation Oncology Department. This should immediately be reported to the management team. Adequate testing and reporting to authorities in positive cases is mandatory. RKI criteria 3 and 4 should be applied to separate patients into those with a less well-founded suspicion: acute respiratory symptoms with/without fever and no stay in regions with COVID-19 cases or clinical/radiological viral pneumonia without alternative diagnosis without exposition risk.
- In patients who are already undergoing radiotherapy and are suspected of having typical COVID-19 symptoms, the treatment should be immediately interrupted, and testing results should be awaited.
- In COVID-19 positive patients, who have not started treatment, it is recommended to postpone treatment initiation whenever medically feasible and does not compromise outcome, survival or quality of life (QoL).
- In SARS-CoV-2 positive patients already undergoing radiotherapy, the continuation of treatment can only proceed when specific measures are taken. Each department should individually weigh their resources and evaluated on a case by cases basis (indication for treatment, performance status of the patient, etc.). If these prerequisites cannot be met, a discontinuation of treatment is mandatory. Especially, as the case load of SARS-CoV-2-positive patients is increasing significantly, a strict no-RT-policy can be decided.
- If treatment continues, these measures must be taken:
  - ○The treatment has to be carried out under maximal safety conditions to guarantee the protection of health professionals.
  - ○The patients should be treated at a special linear accelerator, with a specific access route to prevent contact to other patients. This can for example be performed at the end of the regular treatment schedules.
  - ○The staff needs appropriate personal protection equipment according to institutional guidelines and availability.
  - ○The equipment and linac must be adequately sanitized at the end of treatment.
- Alternatively, all treatments in SARS-CoV-2 positive patients could be interrupted to avoid infections of the staff and other patients
- In SARS-CoV-2 positive patients declared cured from the disease, a careful evaluation has to be performed prior to proceed radiotherapy with less advanced measures.

Taken together, the way we treat cancer in the coming months will change dramatically during this pandemic. While we are used to counselling patients on their best treatment option, we will be confronted with a whole new dimension – where we will have to balance the benefits of oncological therapies against the increased risk of cancer patients for SARS-CoV-2 infections. The indications and timelines of our radiotherapy treatments may shift, and we might accept higher risks of cancer recurrences over a short-term increase in risk of death from COVID-19. Despite the increased anxiety and uncertainty that all cancer patients face while awaiting time-dependent treatments, this could even be worse during these days of social distancing and unavailable treatment options. Please consider this aspect during your clinical routine. The selected and presented scientific evidence, it’s interpretation and translation into radiation oncology specific recommendations by this multi-institutional and international collaboration need to be put into the very special context of the COVID-19 pandemic. Firstly, there is still very little knowledge or even evidence available, yet. Available data is sometimes conflicting and incomplete. This knowledge is luckily expanding at enormous speed, which makes however the generation of a founded synthesis difficult. In such a dynamic situation, this manuscript therefore reflects a snapshot, which may become outdated rapidly. Recommendations aim to maximize cancer care of all patients, best-as possible protection of our health care workers and simultaneously rigid suppression of the pandemic spread. It is obvious that not all three goals can be followed and can be achieved to 100%, compromises in one or the other way will have to be made. Such multi-factorial problems will require difficult decisions: decisions will be influenced by various stakeholder (government, health authorities, hospital and university administration), will be restricted by logistical and financial aspects, will need to follow the respective legal frameworks, will need to be put into the political and cultural context, and will at the same time need to consider the individual patient and their families. Consequently, there will be differences between countries, states, institutions and even between individual clinicians, all trying their best in such situations of crisis. Finally, we do not know, yet, whether the structured planning described in this manuscript will stand or survive the potentially dramatic developments in the future, where irrational or hard actions might become reality.

## Data Availability

All references are cited in the manuscript. There is no additional data and material involved.
